# Lithography-Free Electrical Contact Method for Optoelectronic and Flexible Devices Based on Mechanically Exfoliated 2D Materials

**DOI:** 10.3390/mi17070844

**Published:** 2026-07-16

**Authors:** Paolo Salvemme, Diego Vennarini, Riccardo Frisenda

**Affiliations:** Physics Department, Sapienza University of Rome, Piazzale Aldo Moro 5, 00185 Rome, Italy

**Keywords:** 2D materials, optoelectronics, flexible electronics, electrical contacts, MoS_2_, graphene

## Abstract

We report a tabletop, versatile and lithography-free electrical contacting method for two-dimensional (2D) materials and van der Waals (vdW) heterostructures based on silver paint micromanipulation (SPMM). Operated under an ambient optical microscope, this additive, room-temperature approach circumvents the chemical solvents and high temperatures associated with conventional cleanroom processing used in electrode fabrication. We validate the efficacy of this strategy by fabricating devices based on high-quality mechanically exfoliated thin flakes on both rigid SiO_2_/Si and flexible polycarbonate substrates. On rigid supports, SPMM-contact multilayer graphene devices exhibit linear Ohmic behavior with excellent environmental stability over multiple days and an ambipolar field effect. Gate-tunable multilayer graphene/few-layer MoS_2_/multilayer graphene field-effect transistors demonstrate n-type gating with a two-terminal carrier mobility of $60\;\frac{{\mathrm{cm}}^{2}}{\mathrm{Vs}}$ and time-resolved photoresponse under 660 nm and 415 nm illumination, with responsivities as high as 10 A/W at the lowest incident powers. The SPMM method can also be carried out on flexible polymeric substrates such as polycarbonate, which is notoriously difficult to work with in microfabrication. We demonstrate a flexible multilayer graphene device that functions as highly responsive piezoresistive strain sensors at low deformations with a gauge factor of 50. Finally, a fully integrated flexible vdW photodetector is tested up to 1.2% uniaxial tensile strain. Despite experiencing local micro-fracturing of the MoS_2_ channel, the localized vdW junctions maintain robust charge collection, yielding photodetecting capabilities under tensile strain. This simple and cost-effective electrical contacting technique establishes a highly accessible platform for the rapid prototyping and mechanical testing of next-generation optoelectronics and flexible electronics based on 2D materials and vdW heterostructures.

## 1. Introduction

Since the isolation of graphene (Gr), two-dimensional (2D) materials have attracted much research interest due to their extraordinary electronic, optical, and mechanical properties [1,2,3,4]. Beyond graphene, the emergence of transition metal dichalcogenides (TMDCs), such as MoS_2_ and WSe_2_, has opened new horizons for next-generation optoelectronic applications, including high-performance photodetectors, light-emitting diodes and photovoltaics [5,6,7]. Characterized by their atomic thickness and high mechanical flexibility, these van der Waals crystals are uniquely suited for the field of flexible and wearable electronics, where traditional bulk semiconductors often fail under mechanical strain [8,9,10,11]. In particular, devices based on single atomically thin mechanically exfoliated flakes or van der Waals (vdW) heterostructures are particularly appealing for scientists given their ideal properties and low defect density [12]. Nevertheless, contacting these materials typically requires microfabrication capabilities and cleanroom processing. The high costs and operational complexity of these facilities place them out of reach for many academic and industrial laboratories worldwide. This steep barrier to entry underscores a critical, unmet need for alternative, benchtop-compatible contacting strategies that can help the development of 2D electronics without sacrificing device performance.

A second issue related to the microfabrication of electrodes is compatibility with substrates. While these techniques work very well with conventional rigid substrates such as SiO_2_/Si, they struggle with most of the flexible substrates used in flexible electronics. In fact, while SiO_2_/Si easily tolerate high-temperature baking, metal evaporation and aggressive chemical solvents, flexible polymers, such as polycarbonate (PC), polyimide (PI), or polydimethylsiloxane (PDMS), are vulnerable to these processing conditions [13,14,15,16]. Standard lithographic workflows need resist-baking steps that can approach or exceed the glass transition temperatures of many flexible polymers, inducing thermal warping or outgassing in vacuum chambers. Furthermore, prolonged exposure to organic solvents like acetone, developers and liftoff strippers frequently causes swelling, structural degradation or chemical crazing of the polymer surface. Also, metal evaporation can be problematic due to mismatches in thermal expansion coefficients between the metal and the polymeric substrate leading to high residual stress, resulting in delamination or cracking of electrical contacts upon mechanical flexing. Consequently, realizing the full potential of flexible 2D optoelectronics demands a gentle, room-temperature and chemical-free contact deposition technique.

To overcome the issues described above, efforts in the literature about 2D devices have been dedicated to the development of lithography-free contacting techniques for 2D materials. One early strategy relied on physical shadow masks and microsoldering techniques [17,18,19,20,21,22], which allow for the direct evaporation of metal leads onto the 2D channel material without any polymer resist exposure. While effective at preserving clean channel areas, shadow masks are difficult to fabricate, can have geometric restrictions and still require high-vacuum evaporation systems. A second strategy is the direct microprobing of 2D materials [23,24,25,26]. Here, electrical junctions are established using conductive atomic force microscopy (AFM) tips, microscopic probe needles, nanoribbon microprobes or flexible carbon fiber micro-needles. Although direct microprobing serves as an exceptional tool for rapid, non-destructive transport screening, these mechanical contacts are temporary and highly susceptible to ambient drift. To achieve permanent contacts, automated direct-write printing techniques such as direct ink writing and aerosol-jet printing of liquid metals or silver nanoparticle inks have emerged as highly customizable routes for rapid 2D device prototyping [27,28]. However, automated printing demands specialized equipment, complex ink engineering and high-temperature curing steps that can damage heat-sensitive substrates like polycarbonate. Recently, attaching silver (Ag) paint contacts to a CVD-grown MoS_2_ flake on SiO_2_/Si was demonstrated by Cho and coauthors [29], but the method presented in their article relies on the direct manipulation of Ag paint by hand, making it very difficult to execute precisely and reducing the reproducibility and the device fabrication yield.

In this article, we demonstrate a robust, versatile, and entirely lithography-free contacting method for 2D materials based on silver paint micromanipulation (SPMM) under an optical microscope. This benchtop approach completely bypasses the need for microfabrication, metal evaporation and the use of aggressive solvents. We validate the efficacy of this strategy by successfully fabricating high-performance electrical contacts on single flakes of multilayer (ML) and few-layer (FL) graphene and stacked graphene/MoS_2_/graphene van der Waals heterostructures. We demonstrate functional devices on both conventional rigid SiO_2_/Si and flexible polycarbonate substrates without inducing any thermal deformation or chemical degradation. Electrical measurements reveal linear Ohmic contacts on Ag/ML Gr/Ag devices with good ambient stability over multiple days, alongside gate-modulated field-effect transistors (FETs) showing the ambipolar characteristics typical of Gr. We further demonstrate our technique for optoelectronic devices based on applying Ag/Gr contacts to few-layer MoS_2_. When fabricated on SiO_2_/Si, these devices show n-type transfer characteristics with low hysteresis and good two-terminal mobility up to $60\;\frac{{\mathrm{cm}}^{2}}{\mathrm{Vs}}$. Furthermore, time-resolved optoelectronic characterization under 660 nm and 415 nm illumination confirms that this fabrication route preserves pristine electronic interfaces and high crystal quality. Finally, we demonstrate the fabrication of flexible Ag/ML Gr/FL MoS_2_/ML Gr/Ag devices on PC and test them under strain. Ultimately, this work provides an accessible, non-destructive and low-cost fabrication technique that can be useful for the rapid prototyping of next-generation rigid or flexible 2D electronic and optoelectronic devices.

## 2. Materials and Methods

*Mechanical exfoliation and deterministic transfer of 2D materials.* The fabrication of 2D devices began with the mechanical exfoliation of bulk natural crystals of graphite (ProGraphite GmbH, Untergriesbach, Germany) and molybdenum disulfide (Companheiro No. 1 quarry, Sezures, Penalva do Castelo, Viseu, Portugal). Thin flakes were isolated and exfoliated using a commercial adhesive tape (SPV 224, Nitto Denko, Osaka, Japan) and subsequently transferred onto Gel-Film (WF x4 6.0 mil, Gel-Pak, Hayward, CA, USA) to optimize flake selection via optical microscopy. Selected 2D crystals were then deposited onto the target substrate using a dry deterministic transfer technique performed under ambient conditions. To do this, the Gel-Film stamp is attached to a glass slide and mounted on a 3-axis micromanipulator to transfer the flakes to an arbitrary substrate, also mounted on a second micromanipulator. The rigid substrates consisted of highly doped silicon with a 300 nm thick thermally grown silicon dioxide, while the flexible substrates consisted of polycarbonate sheets with a nominal thickness of 0.25 mm. A long-working-distance microscope allows the transfer process to be directly observed. The macroscopic electrical leads were then deposited via room-temperature silver paint micromanipulation technique, as detailed in Section 3. For all devices fabricated on rigid SiO_2_/Si substrates, immediately following the initial room-temperature solidification of the silver paint (16040-30 Fast Drying Silver Paint, Ted Pella, Redding, CA, USA) in air (which takes approximately 10 min), the samples were transferred to a hot plate and subjected to a post-fabrication thermal annealing step at 90 °C for 30 min under ambient environment.

*Three-point bending apparatus.* The electromechanical characteristics and piezoresistive behavior of the flexible devices on polycarbonate were evaluated by applying controlled uniaxial strain to the active channel areas. Mechanical deformation was applied by flexing the compliant polymer sheets using a custom-built three-point bending apparatus detailed elsewhere [30].

## 3. Results and Discussions

**Figure 1:**
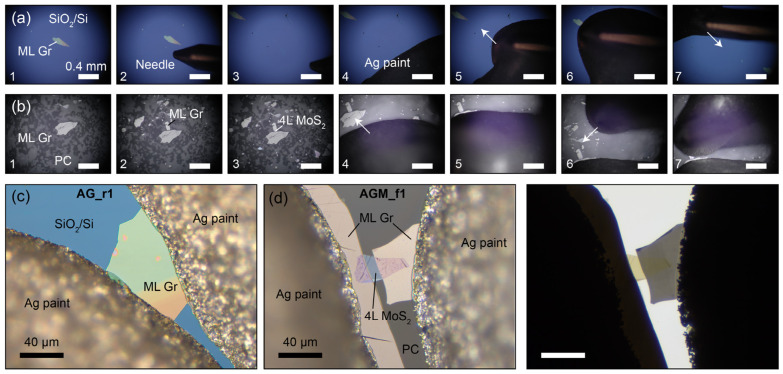
**Lithography-free electrical contacting to 2D flakes.** (**a**) Optical photograph of silver paint micromanipulation showing the probe tip and flake/substrate alignment (**1**–**3**) and the application and manipulation of silver paint (**4**–**7**). The device consists of a single ML Gr flake on SiO_2_/Si. The white arrows indicate the direction of movement of the probe tip. (**b**) Same as (**a**) for a van der Waals heterostructure consisting of ML Gr/4L MoS_2_/ML Gr fabricated onto polycarbonate by sequential transfers (**1**–**3**) and electrical contacting by silver paint (**4**–**7**). (**c**) Optical micrograph of the ML graphene device (AG_r1) fabricated in (**a**). (**d**) Optical micrographs in reflection (**left**) and transmission (**right**) illumination modes of the ML graphene/MoS_2_/ML graphene van der Waals heterostructure (AGM_f1) fabricated in (**b**).

To electrically address mechanically exfoliated 2D crystals without exposing the fragile flakes or heat-sensitive substrates to polymer resists, organic solvents or thermal stresses, we fabricated electrical contacts by SPMM. Figure 1a shows the fabrication of electrical contacts onto a single ML Gr flake on SiO_2_/Si, while Figure 1b shows an ML Gr/4L MoS_2_/ML Gr vdW heterostructure on PC. The fabrication protocol begins with the mechanical exfoliation of the target 2D materials using Nitto tape and Gel-Film followed by the deterministic transfer onto the desired substrate (panel 1, Figure 1a) [31]. After the flake transfer, the substrate is positioned under an optical microscope integrated onto a mechanical micromanipulator capable of spatial translation along the x, y, and z axes. A second micromanipulator carries a metallic microprobe needle that is positioned in the immediate vicinity of the target flake (panels 2 and 3). Next, a micro-droplet of commercial silver paint (16040-30 Fast Drying Silver Paint, Ted Pella, Redding, CA, USA) is deposited directly onto the substrate adjacent to the needle apex (panel 4). Utilizing the micromanipulator stages, the needle is then used as a mechanical guide to physically pull and manipulate the viscous silver paint across the substrate surface (panel 5). The silver paint is moved until it seamlessly wets the targeted edge of the graphene flake, establishing the first electrical contact (panel 6). The microprobe is subsequently retracted, and the exact same micromanipulation sequence is repeated on the opposite side of the flake to complete the two-terminal device architecture (panel 7). Because the silver paint is manipulated in its liquid phase, it naturally conforms to the edge of the 2D material, ensuring a robust physical interface without inducing mechanical tearing or strain in the flake. The process just described is easy to implement and leads to a high reproducibility and fabrication yield (see Figure S8 of the Supporting Information). Furthermore, as demonstrated in Figure 1b, this manipulation technique can be applied also to vdW heterostructures and flexible substrates.

The microscope photograph of the two devices contacted in Figure 1a,b, AG_r1 and AGM_f1, respectively, are shown in Figure 1c,d. In Figure 1c, a single multilayer graphene flake (thickness ~ 120 layers, 120L, see Figures S1 and S2 of the Supporting Information) located on a rigid SiO_2_/Si substrate, with the sharp boundary and clean interface between the liquid-deposited silver contacts and the active channel, is visible. Figure 1d instead shows reflection (left) and transmission (right) illumination mode microscope photographs of a vertically stacked ML Gr/4L MoS_2_/ML Gr vdW heterostructure directly assembled on a flexible polycarbonate substrate. Despite the high chemical vulnerability of polycarbonate to conventional cleanroom solvents like acetone, the PC substrate retains its pristine structural integrity and exceptional optical transparency, entirely free from the chemical crazing, swelling, or thermal warping typically induced by conventional lithographic microfabrication.

**Figure 2:**
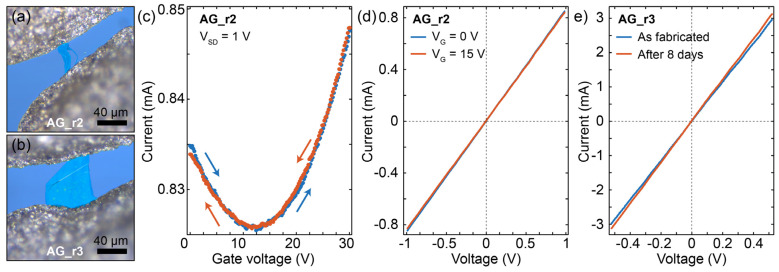
**Electrical characterization of lithography-free multilayer graphene devices.** (**a**,**b**) Optical micrographs of two representative multilayer graphene devices (AG_r2 and AG_r3) fabricated on SiO_2_/Si substrates using micromanipulated silver paint contacts. (**c**) Transfer characteristic of device AG_r2 measured at a constant bias $V_{SD}=\;1\;V$. Arrows indicate the forward (blue circles) and backward (orange circles) gate sweep directions. (**d**) Current–voltage characteristics of device AG_r2 recorded at gate voltages of $V_{G}=\;0\;V$ (blue curve) and 15 V (orange curve). (**e**) Long-term environmental stability of device AG_r3, comparing the IV characteristics measured immediately after fabrication (blue curve) and after 8 days of continuous storage under ambient atmospheric conditions (orange curve).

To evaluate the electrical quality of the SPMM contacts, two-terminal transport measurements were performed on few-layer and multilayer graphene devices fabricated on rigid SiO_2_/Si substrates. Figure 2a,b display optical micrographs of two representative devices, denoted as AG_r2 (flake thickness ~ 9L, see Figure S3 of the Supporting Information) and AG_r3 (flake thickness ~ 29L, see Figure S4 of the Supporting Information), respectively, featuring well-defined silver electrodes bridging the isolated graphene channels. The highly doped silicon substrate was utilized as a global back-gate to modulate the carrier density in the thinnest graphene channel. The field-effect transport properties of device AG_r2 are presented in Figure 2c, which shows the transfer characteristic (source-drain current $I_{SD}$ versus back-gate voltage $V_{G}$) acquired at a constant source-drain bias of $V_{SD}=\;1\;V$. The device exhibits a clear ambipolar transport modulation with a charge neutrality point (minimum conduction) located at a positive gate voltage $V_{G}=\;12.5\;V$, indicating a moderate level of intrinsic p-type doping. This behavior is typical for unencapsulated exfoliated graphene processed in ambient conditions and is primarily attributed to the adsorption of atmospheric oxygen and water molecules [32,33]. The transfer curve displays minimal hysteresis between the forward and backward gate voltage sweeps, suggesting a relatively clean van der Waals interface free from trapped mobile ions or contamination. From the transfer curve, we extracted the two-terminal mobility according to the following formula:

(1) $$\mu =\frac{L}{WC_{ox}V_{SD}}\frac{\partial I_{SD}}{\partial V_{G}},$$

where L≈30 μm and W≈10 μm are the length and width of the channel, and $C_{ox}=11.6\;\mathrm{nF}/{\mathrm{cm}}^{2}$ is the capacitance per unit area of 300 nm thick SiO_2_. We found that $\mu \approx 780\;\frac{{\mathrm{cm}}^{2}}{\mathrm{Vs}}$, which is typical of FL graphene where charged impurities, surface optical phonons in the SiO_2_ and surface roughness restrict the overall carrier transport [34,35]. Note that the mobility estimated from Equation 1 is a two-terminal mobility, which can include contributions from contact resistance and contact gating apart from channel gating [36].

Figure 2d shows the IV characteristics for device AG_r2 measured at gate voltages of $V_{G}=\;0\;V$ and $V_{G}=\;15\;V$. In both states, the current–voltage sweeps are linear, confirming the formation of Ohmic contacts between the micromanipulated silver paint and the 9L graphene flake. The high drive currents, which reach a density of about $2.4\;\mathrm{mA}/{\mu m}^{2}$, further reflect a high-quality electrical interface with low contact resistance. An estimation of the width-normalized contact resistance of SPMM-fabricated electrodes on multilayer graphene is shown in Figure S6 of the Supporting Information. From the transfer length method applied to six devices with channel thicknesses between 30 nm and 50 nm, we found a width-normalized contact resistance equal to (90 ± 30) Ω µm, comparable to state-of-the-art lithographically fabricated electrodes [37,38,39].

Also, the time stability of the fabricated contacts is excellent. A frequent limitation of alternative, non-lithographic contacting techniques is their susceptibility to rapid ambient degradation or mechanical instability. To demonstrate the robust reliability of our method, we monitored the electrical performance of device AG_r3 over extended periods. Figure 2e compares the output characteristics of the device immediately after fabrication and after 8 days of continuous exposure to ambient atmospheric conditions. The conductance of the device remains constant, with no significant degradation in current magnitude or deviation from Ohmic linearity. Similar results were found for three additional devices, showing good stability over the course of 250 h, as shown in Figure S7 of the Supporting Information. This temporal stability indicates that the silver paint contacts cure to form a physically robust and chemically stable metallic junction, making this technique highly suitable for long-term device testing and practical benchtop prototyping. Overall, the three devices AG_r1, AG_r2 and AG_r3 have resistances of 100 Ω, 220 Ω and 1150 Ω, respectively.

**Figure 3:**
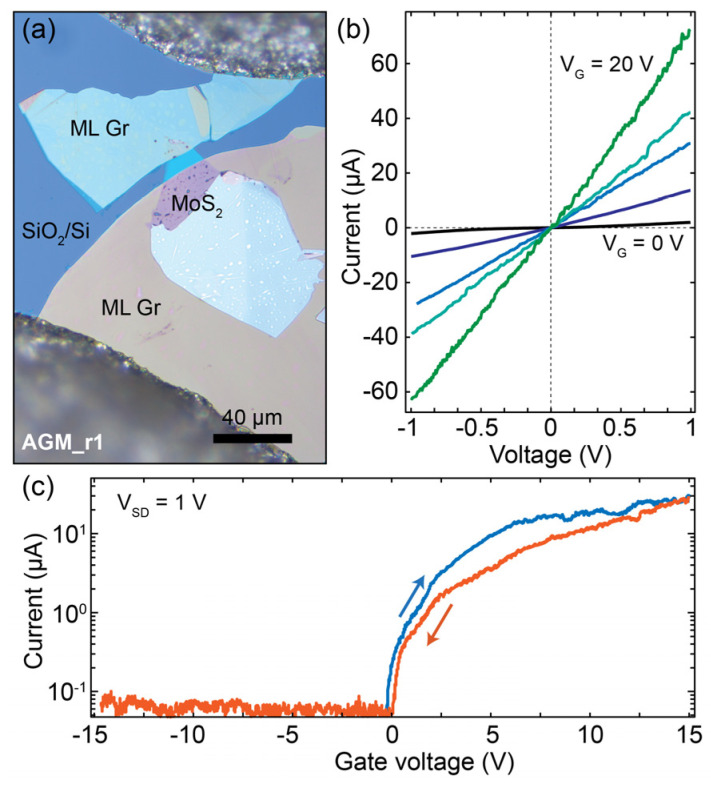
**Electrical transport properties of a lithography-free van der Waals heterostructure field-effect transistor.** (**a**) Optical micrograph of the assembled heterostructure device (AGM_r1) on SiO_2_/Si. The active channel consists of a five-layer MoS_2_ flake contacted by two distinct multilayer graphene flakes, which are connected to macroscopic silver paint electrodes. (**b**) Current–voltage characteristics measured at different back-gate voltages ranging from 0 V to 20 V in steps of 5 V. (**c**) Transfer characteristic recorded at a constant source-drain bias of 1 V. The blue and orange arrows indicate the forward (blue curve) and backward (orange curve) gate voltage sweep directions.

To demonstrate that our lithography-free contacting method is fully compatible with vdW heterostructures, architectures, and semiconducting optoelectronics, we fabricated a vertically stacked vdW FET. Here, we utilized a ML graphene-contacted geometry where multilayer graphene flakes serve as atomically smooth, low-resistance intermediate electrodes between the active MoS_2_ channel and the macroscopic silver paint pads [40,41,42,43,44]. Figure 3a displays an optical micrograph of the completed heterostructure device, designated as AGM_r1, assembled on a standard SiO_2_/Si substrate. The channel consists of a thin, exfoliated five-layer (5L, see Figure S5 of the Supporting Information) MoS_2_ flake bridging two distinct ML Gr sources and drain flakes, which were previously transferred onto the substrate. The IVs of the device under various global back-gate voltages are presented in Figure 3b. The device demonstrates efficient gate-modulated charge transport; at $V_{G}=\;0\;V$ (black curve), the device is in a highly resistive OFF state, with a low-bias resistance of 960 kΩ. As $V_{G}$ is increased to 20 V (green curve), the source-drain current increases, and the device resistance decreases to 14 kΩ. This behavior confirms an n-type field-effect conduction mechanism, which is characteristic of electron-doped, thin MoS_2_ flakes. The IV curves display excellent symmetry and evolve from slightly non-linear characteristics at low gate biases into linear regimes at higher positive gate voltages.

To further quantify the gate-modulated transport in AGM_r1, a transfer characteristic was recorded at a constant source-drain bias of $V_{SD}=\;1\;V$ and shown in Figure 3c on a semi-logarithmic scale. The FET exhibits a sharp n-channel turn-on behavior with a threshold voltage close to $V_{G}=\;0\;V$. Below the threshold, the device turns off, with currents down to approximately 60 nA, while above 0 V, the current increases by nearly three orders of magnitude. Using Equation 1, we extracted a mobility of $\mu \approx 60\;\frac{{\mathrm{cm}}^{2}}{\mathrm{Vs}}$, which is a value larger than the mobilities typically extracted in FL MoS_2_ contacted by conventional metallic electrodes such as Au/Ti [45,46,47]. Additionally, moderate clockwise hysteresis is observed between the forward (blue curve) and backward (orange curve) gate voltage sweeps. This hysteretic behavior can be attributed to charge trapping and detrapping processes associated with atmospheric adsorbates or intrinsic and interface traps typical in unencapsulated TMD transistors operated under ambient conditions [48,49,50,51]. The realization of an atomically thin field-effect transistor via benchtop fabrication highlights the structural integrity of the vdW interfaces preserved by our gentle, solvent-free contacting technique.

**Figure 4:**
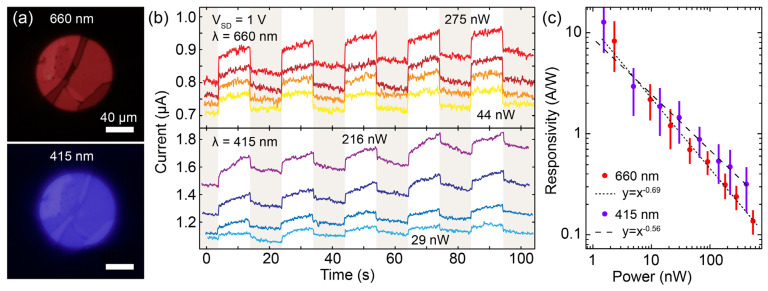
**Optoelectronic performance of the graphene/MoS_2_/graphene heterostructure.** (**a**) Optical micrographs of AGM_r1 device under global illumination at wavelengths of 660 nm (**top**) and 415 nm (**bottom**). (**b**) Time-resolved photoresponse recorded at a bias of 1 V under alternating dark and light cycles for 660 nm (**top**) and 415 nm (**bottom**) wavelengths. Shaded vertical regions indicate the periods of optical illumination. Successive cycles shown with different colors correspond to increasing incident optical power densities. The curves have not been vertically offset, but the difference in dark currents comes from a persistent photocurrent effect. (**c**) Log–log plot of the responsivity as a function of the incident power for the 660 nm (red data points) and 415 nm (purple data points) excitations. The dashed lines represent power-law fits to the data. The error bars have been calculated by standard deviations from multiple switching cycles.

After the characterization of field-effect transport of AGM_r1 kept in dark, we investigated the optoelectronic performance of the device. The sample was studied under global illumination using two distinct excitation wavelengths, 660 nm (M660FP1, Thorlabs, Newton, NJ, USA) and 415 nm (M415F3, Thorlabs, Newton, NJ, USA), both of which provide photon energies exceeding the bandgap of the 5L MoS_2_ channel. Figure 4a displays the optical micrographs of the device illuminated by the two light sources, using a circular spot with a diameter of approximately 110 μm, ensuring that the entire channel area between the graphene electrodes is uniformly illuminated without illuminating the Ag/ML graphene interface. The time-resolved photoresponse of the device was recorded at a constant source-drain bias of $V_{SD}=\;1\;V$. Figure 4b shows the device current as a function of time recorded while turning ON and OFF the light source at different powers for both the 660 nm (top panel) and 415 nm (bottom panel) excitation. Upon illumination, the device exhibits a rapid increase in photocurrent followed by a slow increase. When the optical excitation is removed, the current does not immediately return to its initial dark state but exhibits a fast initial drop followed by a prolonged, slow decay. This leads to a measurable persistent photocurrent (PPC), which manifests as an upward vertical offset in the baseline dark current over subsequent illumination cycles in Figure 4b. These results are consistent with localized trap states preventing immediate recombination and resulting in a prolonged excess electron density in the channel after the light is turned off [52,53,54].

To quantify the photodetection efficiency, we extracted the responsivity R of the device, defined as $R=I_{Ph}/P$, where $I_{Ph}$ is the generated photocurrent, and P is the incident optical power on the MoS_2_ channel. Figure 4c presents the responsivity as a function of incident power on a log–log scale for both 660 nm and 415 nm excitations. The responsivity reaches maximum values larger than 10 A/W at the lowest measured optical powers and decreases at higher powers. The data for both wavelengths are well described by a power-law fit $R=P^{\alpha }$, with an extracted exponent α=−0.69 for 660 nm and α=−0.56 for 415 nm excitation. The observed sub-linear scaling can be assigned to photogating and trap-assisted recombination mechanisms commonly observed in thin transition metal dichalcogenides [55,56]. This photogeneration mechanism is also consistent with the large responsivity observed at low powers, which require the presence of photogain. Notably, because the device operates without an encapsulation layer, the exposed MoS_2_ surface is susceptible to ambient gaseous adsorbates, which could play a role in the photogeneration mechanism. These results confirm that our lithography-free processing method successfully preserves the native semiconducting and optoelectronic properties of 2D semiconducting flakes.

**Figure 5:**
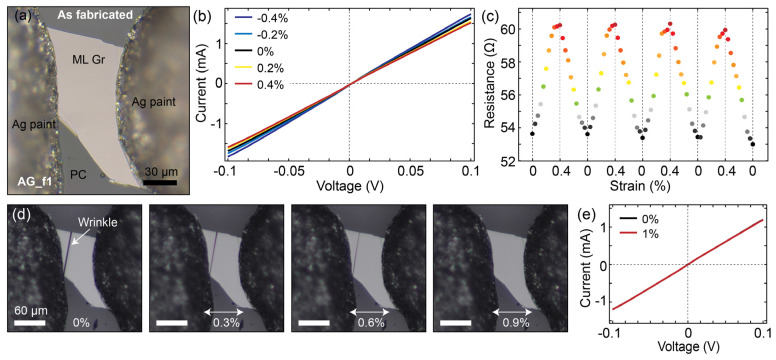
**Electrical transport and strain-tuning of multilayer graphene on a flexible substrate.** (**a**) Optical micrograph of the multilayer graphene device (AG_f1) fabricated directly on a 0.25 mm thick flexible polycarbonate (PC) substrate using micromanipulated silver paint contacts. (**b**) Current–voltage characteristics under small applied uniaxial strains ranging from −0.4% (compression, blue) to 0.4% (tension, red). (**c**) Resistance at 0.01 V of bias during cyclical loading between 0% and 0.4% strain. (**d**) Sequential optical micrographs tracking the structural evolution of the graphene channel under increasing tensile strain (0% to 0.9%) after the formation of a wrinkle highlighted by the white arrow. (**e**) Current–voltage characteristics recorded at 0% and 1% strain in the presence of the wrinkle from (**d**).

To demonstrate the versatility of our tabletop, solvent-free contacting method for flexible electronics, we moved from rigid silicon to compliant polymer substrates. Conventional cleanroom lithography on polymers is notably difficult due to chemical incompatibility with standard solvents and restricted thermal processing windows. Our direct-write silver paint technique circumvents these limitations, enabling room-temperature device fabrication directly onto chemically sensitive polymer sheets. Figure 5a shows an optical micrograph of a representative two-terminal multilayer graphene device, designated as AG_f1, fabricated on a 0.25 mm thick flexible polycarbonate substrate. Uniaxial tensile and compressive strain ε was systematically applied to the channel using a custom-built three-point bending apparatus [30]. The electrical response under low uniaxial strain conditions (±0.4%, where positive values correspond to tensile strain and negative to compressive strain) is presented in Figure 5b. The strain ε is calculated according to $\varepsilon =\frac{6td}{L^{2}}$, where t is the substrate thickness, L=20 mm is the distance between the external pillars of the setup, and d is the controlled displacement of the central pillar in the vertical direction. The quantity ε represents an upper value for the strain applied to the 2D channel, since the formula gives the uniaxial strain on the surface of polycarbonate. The IV characteristics of AG_f1 are linear for both tensile and compressive regimes, confirming that the mechanical deformation of the underlying polymer does not degrade the Ohmic contacts to ML Gr or cause delamination of the silver paint electrodes. Figure 5c shows the change in resistance R for multiple tensile strain cycling sequences, which is reproducible and reversible. When tensile strain is applied, the resistance increases, and when releasing the strain back to 0%, the resistance completely recovers to its initial value of about 53.5 Ω. From this low-strain regime, we extracted a gauge factor $GF\;=\frac{R-R_{0}}{\varepsilon }\approx 50$, where $R_{0}$ is the resistance at 0% strain, demonstrating that the device functions as a highly responsive, reversible piezoresistive strain sensor.

In contrast, a distinct physical regime emerges when the device is subjected to larger mechanical deformations. After applying a strain of ±2%, we observed the formation of a localized structural wrinkle in the ML Gr channel orthogonal to the uniaxial strain axis. As captured in the sequential optical micrographs of Figure 5d, taken at increasing tensile strain values from the new wrinkled configuration at 0% strain, this wrinkle acts as a mechanical bellows, introducing local slack into the 2D material. Consequently, when further external tensile stress is applied to the polycarbonate substrate, the strain is entirely accommodated by the dynamic flattening of this physical wrinkle rather than being transferred to the graphene crystal lattice. In fact, the energy required for out-of-plane bending or unfolding a 2D material is orders of magnitude lower than that required for in-plane lattice stretching. Therefore, the applied macroscopic strain exclusively drives the geometric unfolding of the wrinkle, avoiding the deformation of the carbon lattice, which would induce changes in the electronic and transport properties. As a direct electronic consequence of this structural change, the device becomes insensitive to higher tensile strain fields. This behavior is clearly confirmed by the overlapping IV characteristics displayed in Figure 5e, where the device exhibits nearly identical electronic transport at 0% and 1% strain. The ability to switch from a high-sensitivity strain sensor at low deformations to a strain-invariant device at high deformations highlights the unique mechanical robustness and adaptive nature of our lithography-free contacts to 2D devices on flexible substrates.

**Figure 6:**
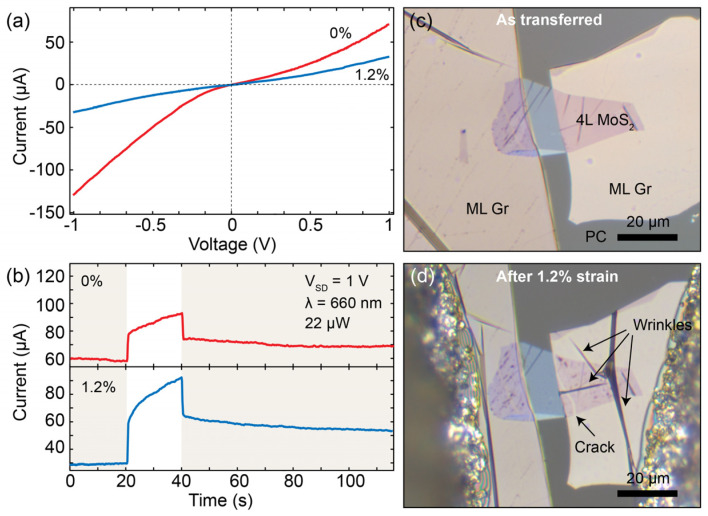
**Flexible photodetector based on lithography-free contacted vdW heterostructure.** (**a**) Current–voltage characteristics of device AGM_f1 based on ML graphene/4L MoS_2_/ML graphene on a flexible polycarbonate substrate recorded at 0% strain (red curve) and under 1.2% uniaxial tensile strain (blue curve). (**b**) Time-resolved photoresponse under alternating 660 nm laser illumination (22 µW incident power) at a bias of 1 V at 0% strain (top) and 1.2% strain (bottom). The shaded areas highlight the dark periods. (**c**,**d**) Optical microscope photographs of the device recorded immediately after fabrication (**c**) and after the application of 1.2% tensile strain (**d**). The arrows highlight the formation of stress-relieving wrinkles and a localized micro-crack across the 4L MoS_2_ channel.

The final device that we demonstrate in this work is a flexible photodetector based on 4L MoS_2_ contacted by two multilayer graphene electrodes fabricated onto polycarbonate. Figure 6a compares the IV characteristics of the flexible heterostructure device (AGM_f1) on a polycarbonate substrate at 0% strain (red curve) and under a uniaxial tensile strain of 1.2% (blue curve). In the unstrained state, the device exhibits currents exceeding 50 µA at 1 V. However, upon bending the substrate to a tensile strain of 1.2%, the source-drain current drops across the entire voltage range, and the IV profile evolves from an asymmetric shape into a more symmetric curve. Despite the reduction in dark conductance upon strain, the device maintains its optoelectronic functionality. Figure 6b presents the time-resolved current measurements under an alternating 660 nm optical excitation at incident power 22 µW at both 0% strain (top panel) and 1.2% strain (bottom panel). At both strain values, the device shows a fast rise in the current upon illumination followed by a slower rise, similar to what is reported for device AGM_r1. After the light is turned off, the current decays fast and then slowly. Interestingly, the responsivity of the device is larger under tensile strain than when unstrained, going from 1.0 A/W at 0% strain to 1.5 A/W at 1.2% strain. Finally, Figure 6c and Figure 6d show the optical microscope photographs of the device just after fabrication and after the application of 1.2% tensile strain, respectively. After the application of strain, we observed the appearance of wrinkles in the ML graphene electrodes similar to those observed in the pure ML graphene device (Figure 5). The 4L MoS_2_ channel, on the other hand, presents a crack across the right ML Gr electrode. This crack, and the weaker mechanical region connected to it, could be at the origin of the large positive gauge factor observed in this device. Nevertheless, the retention of a photoswitching response and photocurrent generation signatures under mechanical deformations indicate that clean van der Waals interfaces remain functional. This highlights the potential of graphene-mediated contacts to preserve optoelectronic performance even in presence of partial mechanical failure of the channel.

## 4. Conclusions

In this work, we have demonstrated the successful realization of advanced electronic and optoelectronic devices based on mechanically exfoliated 2D materials and their van der Waals heterostructures entirely outside of a cleanroom environment using silver paint micromanipulation. By shifting the fabrication methods from lithography and vacuum metal evaporation to a benchtop technique that can be easily implemented in laboratories, the method demonstrated in this manuscript will accelerate the study of 2D materials-based devices.

## Data Availability

The data that support the findings of this study are available from the corresponding author upon reasonable request.

## Supplementary Materials

The following supporting information can be downloaded at: https://www.mdpi.com/article/10.3390/mi17070844/s1, Figure S1: Reflectance spectrum of SiO_2_/Si substrate; Figure S2: Reflectance of multilayer graphene of device AG_r1; Figure S3: Reflectance of multilayer graphene of device AG_r2; Figure S4: Reflectance of multilayer graphene of device AG_r3; Figure S5: Reflectance of few-layer MoS_2_ of device AGM_r1; Figure S6: Contact resistance characterization of Ag/multilayer graphene; Figure S7: Time stability of multilayer graphene devices; Figure S8: Fabrication of five consecutive multilayer graphene devices; Table S1: Device parameters of six SPMM contacted multilayer graphene flakes.
