# Supplementary material for: Lithography-Free Electrical Contact Method for Optoelectronic and Flexible Devices Based on Mechanically Exfoliated 2D Materials
Source: Micromachines (Basel). 2026 Jul 16;17(7):844. doi: 10.3390/mi17070844 (PMC13413952; doi:10.3390/mi17070844)
Supplement: Supplementary file 1 [file micromachines-17-00844-s001.zip › micromachines-4391441-supplementary.pdf]

micromachines

Supporting Information: Lithography-free electrical contact method for optoelectronic and flexible devices based on mechanically exfoliated 2D materials

Paolo Salvemme[1], Diego Vennarini[1] and Riccardo Frisenda[1,*]

[1] Physics Department, Sapienza University of Rome, Piazzale Aldo Moro 5, 00185 Rome, Italy

*E-mail: riccardo.frisenda@uniroma1.it

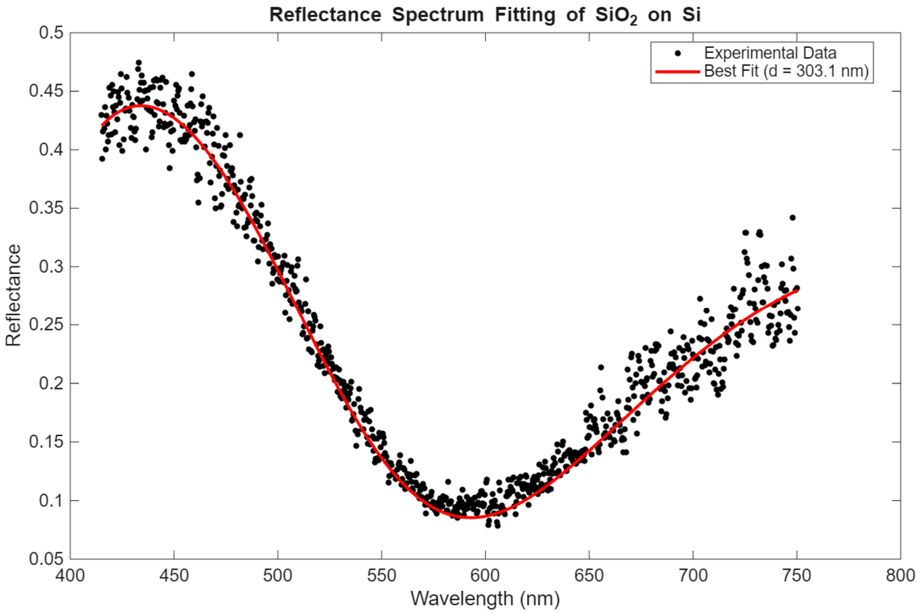

Figure S1. Reflectance of the SiO_2/Si substrate used in the main text and fit to a thin film reflectance model. The thickness extracted for the SiO_2 is 303.1 nm.

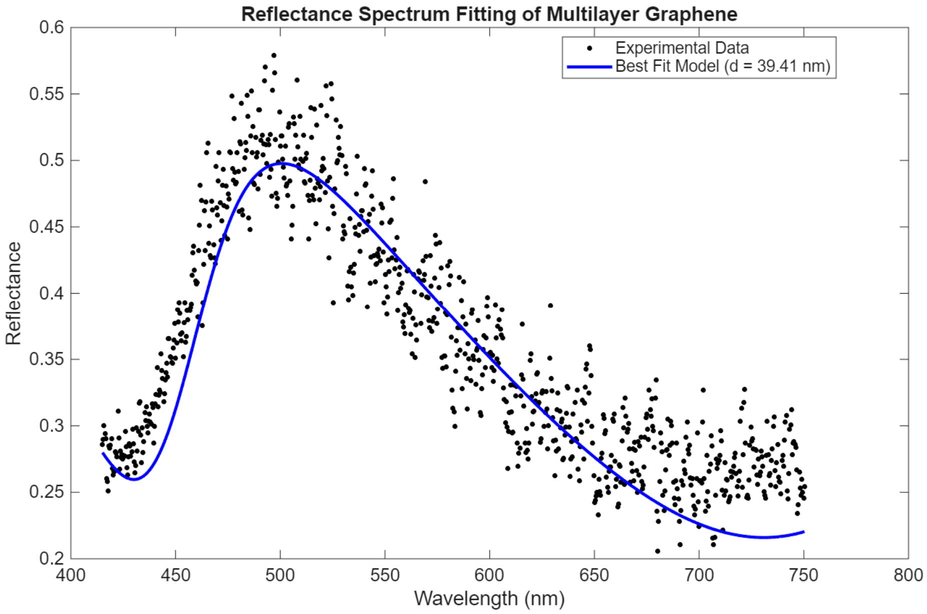

Figure S2. Reflectance of the multilayer graphene used in device AG_r1 and fit to a thin film reflectance model. The thickness extracted is 39.4 nm or 118 layers.

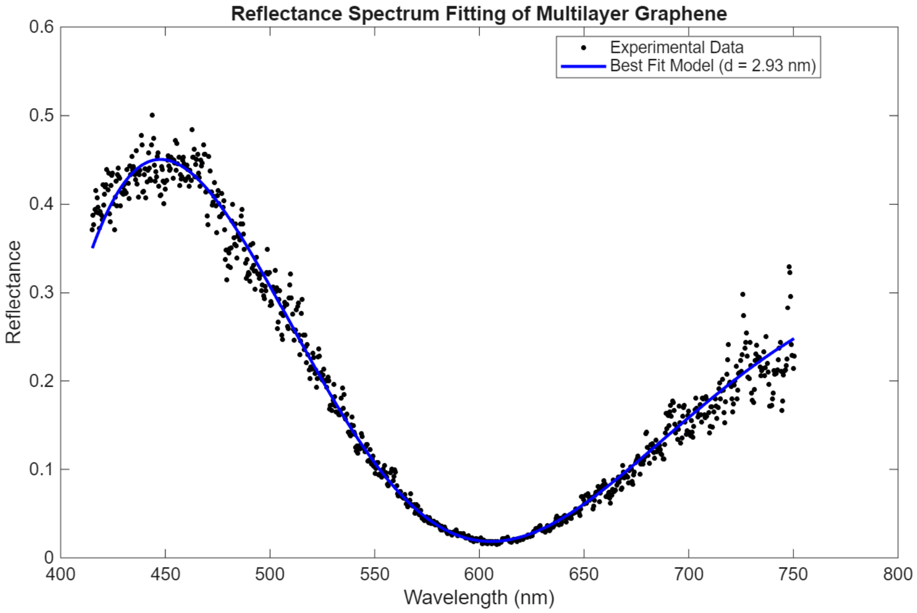

Figure S3. Reflectance of the multilayer graphene used in device AG_r2 and fit to a thin film reflectance model. The thickness extracted is 2.9 nm or 9 layers.

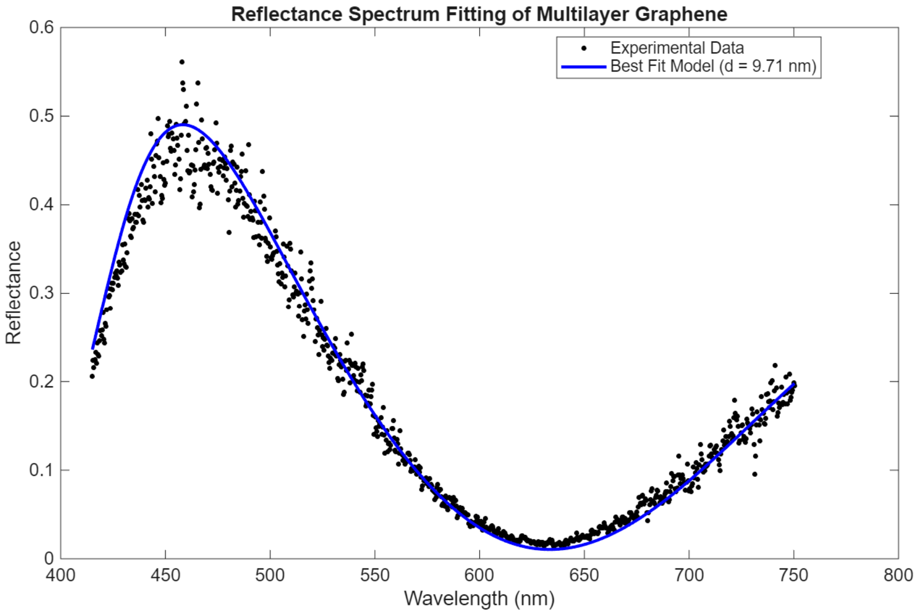

Figure S4. Reflectance of the multilayer graphene used in device AG_r3 and fit to a thin film reflectance model. The thickness extracted is 9.7 nm or 29 layers.

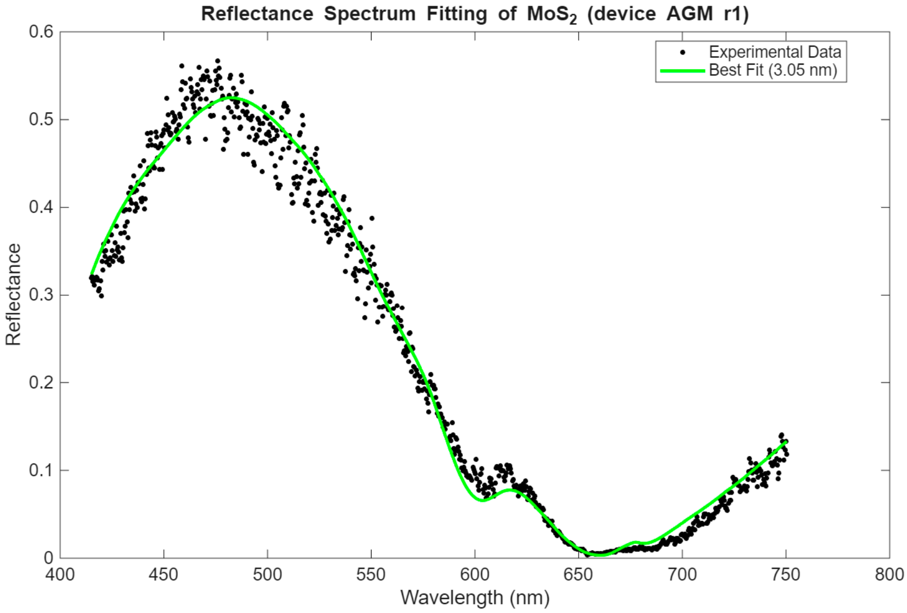

Figure S5. Reflectance of the few-layer MoS_2 used in device AGM_r1 and fit to a thin film reflectance model. The thickness extracted is 3.1 nm or 5 layers.

https://doi.org/10.3390/mi17070844

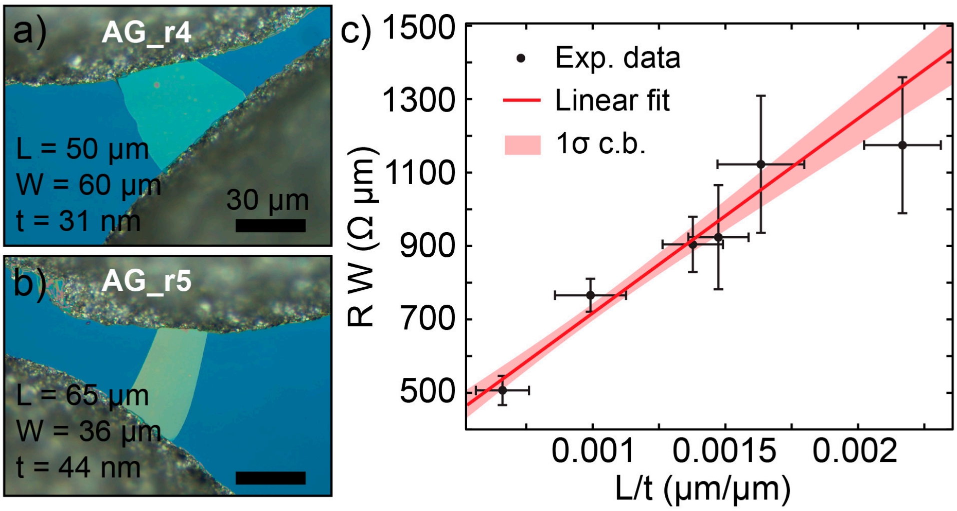

Figure S6. a-b) Optical micrographs of two representative multilayer graphene devices (AG_r4 and AG_r5) fabricated on SiO_2/Si substrates using micro-manipulated silver paint contacts. The length, width and thickness estimated for each device are given in the figure. c) Resistance times channel width as a function of channel length divided by thickness for six devices. Each data point is associated with an error bar calculated by error propagation from the estimated geometrical parameters. The red line is a weighted linear regression to the data and the shaded region corresponds to a one standard deviation confidence band.

**Device**	**Channel length (μm)**	**Channel width (μm)**	**Channel thickness (nm)**	**Resistance (Ω)**
**AG_r4**	50 ± 5	60 ± 10	30.6 ± 0.5	18.7 ± 0.1
**AG_r5**	65 ± 5	36 ± 3	44.0 ± 0.5	25.1 ± 0.1
**AG_r6**	65 ± 5	130 ± 20	44.1 ± 0.5	7.1 ± 0.1
**AG_r7**	33 ± 5	38 ± 3	50.0 ± 0.5	13.3 ± 0.1
**AG_r8**	37 ± 5	85 ± 5	37.3 ± 0.5	9.0 ± 0.1
**AG_r9**	75 ± 5	19 ± 3	34.6 ± 0.5	61.8 ± 0.1

Table S1. Device parameters of six SPMM contacted multilayer graphene flakes.

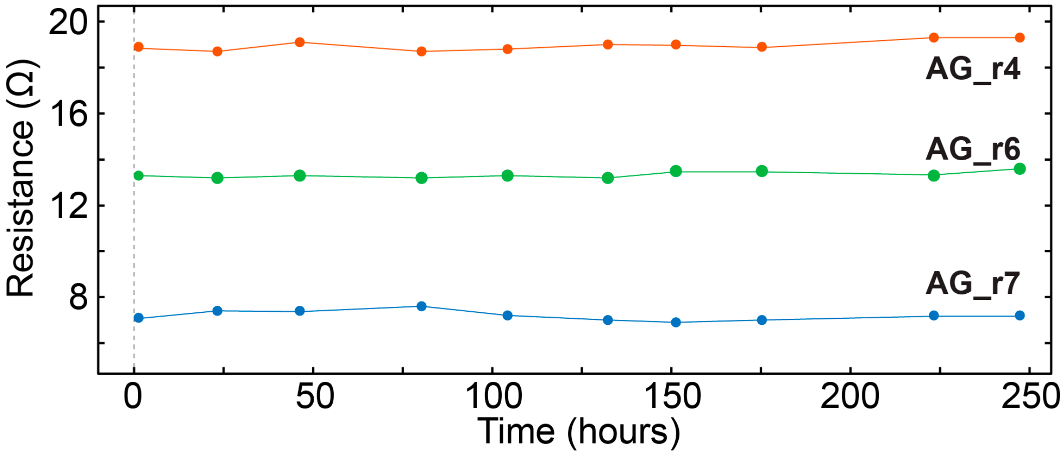

Figure S7. Time stability of three multilayer graphene devices fabricated at time 0 and stored in air and ambient conditions between the different measurements of the resistance.

https://doi.org/10.3390/mi17070844

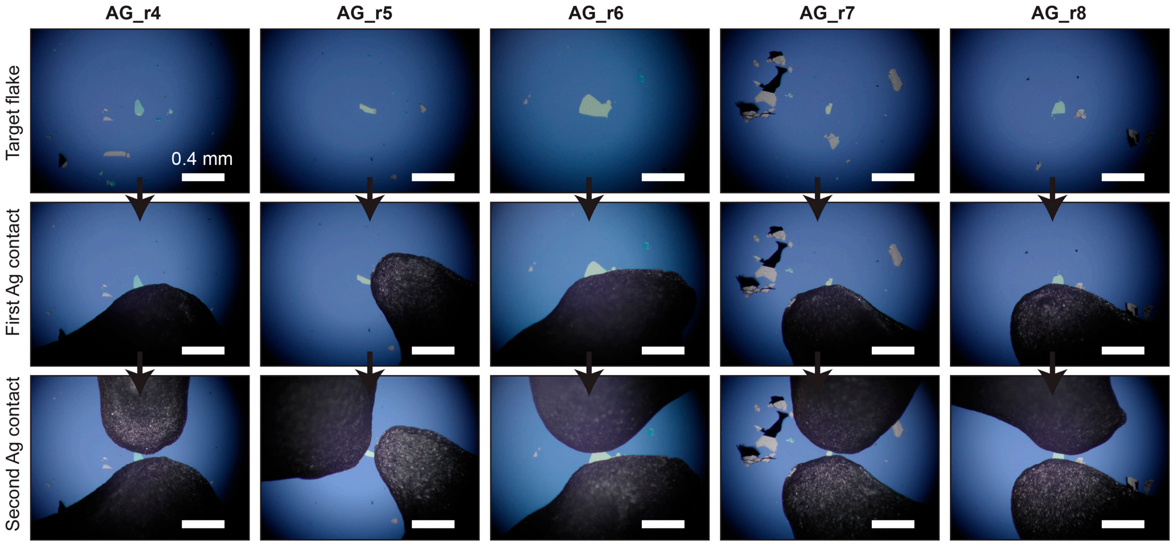

Figure S8. Fabrication of five consecutive multilayer graphene devices. For each device we show the microscope photograph of the target flake (top row), the fabrication of the first electrode (middle row) and of the second electrode (bottom row).
